# Recycling of Electrical Cables—Current Challenges and Future Prospects

**DOI:** 10.3390/ma16206632

**Published:** 2023-10-10

**Authors:** Maciej Wędrychowicz, Jagoda Kurowiak, Tomasz Skrzekut, Piotr Noga

**Affiliations:** 1 Faculty of Mechanical Engineering, Institute of Materials and Biomedical Engineering, University of Zielona Gora, Prof. Z. Szafrana 4 Street, 65-516 Zielona Gora, Poland; j.kurowiak@iimb.uz.zgora.pl; 2 Faculty of Non-Ferrous Metals, AGH University of Science and Technology, 30 Mickiewicza Ave., 30-059 Krakow, Poland; skrzekut@agh.edu.pl (T.S.); pionoga@agh.edu.pl (P.N.)

**Keywords:** plastic materials, recycling/recovery, mechanical processing, copper

## Abstract

Civilization and technical progress are not possible without energy. Dynamic economic growth translates into a systematic increase in demand for electricity. Ensuring the continuity and reliability of electricity supplies is one of the most important aspects of energy security in highly developed countries. Growing energy consumption results not only in the need to build new power plants but also in the need to expand and increase transmission capacity. Therefore, large quantities of electric cables are produced all over the world, and after some time, they largely become waste. Recycling of electric cables focuses on the recovery of metals, mainly copper and aluminum, while polymer insulation is often considered waste and ends up in landfills. Currently, more and more stringent regulations are being introduced, mainly environmental ones, which require maximizing the reduction in waste. This article provides a literature review on cable recycling, presenting the advantages and disadvantages of various recycling methods, including mechanical and material recycling. It has been found that currently, there are very large possibilities for recycling cables, and intensive scientific work is being carried out on their development, which is consistent with global climate policy.

## 1. Introduction

**Figure 1:**
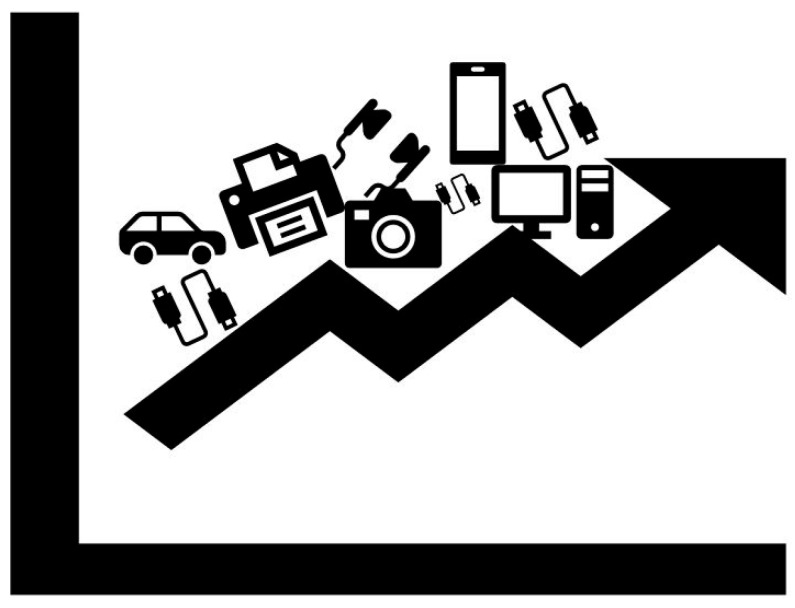
Type of e-waste generated by each section of society.

The contemporary world is grappling with numerous issues, and one of them is the growing number of electronic waste, commonly known as e-waste. The concerns and anxiety associated with this issue are justified. The problem of e-waste is serious because disposal or recycling is costly, and the lack of action and long-term storage of waste in landfills poses a negative impact on the environment, groundwater, and consequently, human health and the lives of other living organisms. It is necessary to seek solutions for processing electronic waste accumulated in landfills because storing them serves no purpose, and their natural decomposition will take decades if not centuries [1]. Due to the continuous development of the economy and industry, the amount of e-waste will continue to rise (Figure 1). Reasons for this include equipment failures rendering them unusable, increased consumption, discarding of obsolete devices, and the ever-advancing technological progress that rapidly introduces new electronic appliances and gadgets to the market [2].

**Figure 2:**
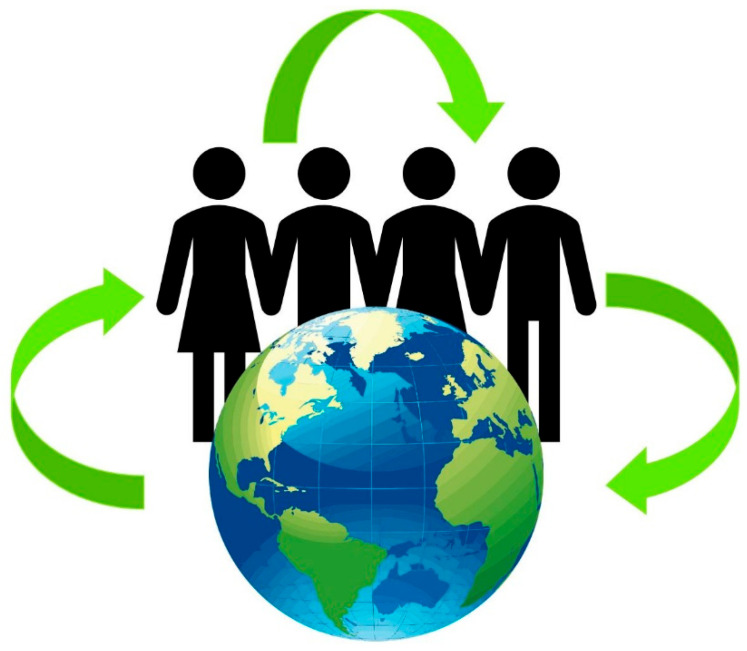
The significance of recycling for people and the environment. Recycling-friendly cycle.

Proper waste management is one of the primary priorities of public institutions dedicated to environmental protection. Waste management systems encompass a wide range of activities aimed at recovering and recycling materials, treating waste as valuable resources (Figure 2). Depending on the type of electronic waste, they vary in structure and composition. Most often, they consist of mixtures of plastics and other chemical compounds, including those classified as hazardous and toxic. Uncontrolled exposure to such substances during their long-term (sometimes illegal) landfilling has harmful effects on human health, soil, water bodies, and the air [3]. Therefore, the primary goal is to prevent these problems and reduce production costs for various products such as metals, plastics, glass, and paper. Recycling clean and homogeneous waste is relatively straightforward, but challenges arise when waste consists of different materials [4,5].

In households, we use a variety of different electrical and electronic devices, such as vacuum cleaners, washing machines, refrigerators, gadgets, mobile phones, computers, and televisions. All of these devices contain cables. Currently, due to the rapid development of energy and information technology industries, as well as changes in consumption habits, the lifespan of electronic equipment ranges from one to six years [6,7]. As a result, electronic equipment (including cables) that was once purchased at a high cost is either sold for scrap or consciously discarded because evolving technology is presented in a more cost-effective manner. Electrical installations in residential buildings, as well as in older family homes that do not meet current safety standards, undergo replacement with new ones, contributing to an increase in the amount of discarded wiring [8].

**Figure 3:**
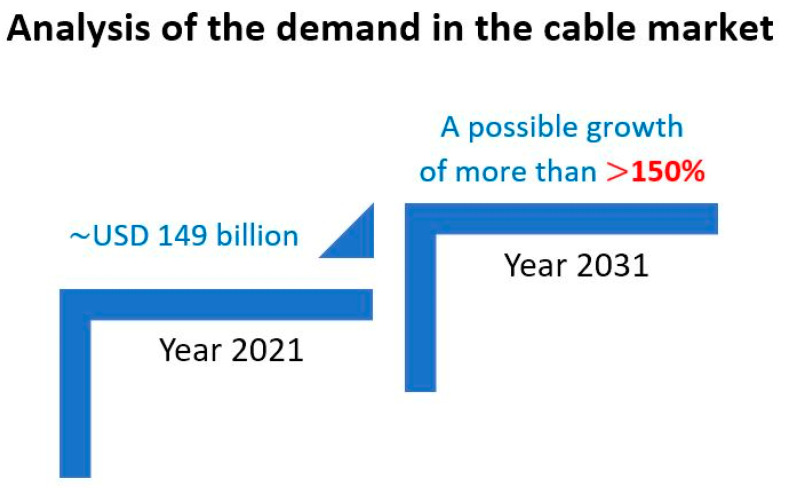
The valuation of the global cable market in 2021 and its forecasted value in 2031. Based on [15].

The global market for electronic and electrical equipment recycling is projected to reach USD 65.8 billion by 2026. Meanwhile, the power cable market is expected to reach USD 277.8 trillion by 2031 (Figure 3), with electronic waste management and recycling accounting for only 17.4% [9].

Aluminum and copper are key products of cable recycling. Global copper production in Asia and Australia alone reached 15.85 million tons in 2017 [10]. The recycling rate of aluminum increased from 45% in 1980 to 69% in 2019 [11]. It is estimated that by 2030, approximately 50% of aluminum will be subjected to recycling [12]. It is widely accepted that recycling aluminum can save around 95% of the energy required for primary aluminum production and reduce greenhouse gas emissions by 97% compared to the primary production process [13]. According to the Bureau of International Recycling (BIR), the production of one ton of aluminum from recycling only requires 12% of the energy compared to primary production [14].

The growth of the electrical cable market is influenced by a number of important variables, including the increasing use of renewable energy sources. Another driving factor for the demand for electrical cables is the expansion of new infrastructure for so-called “smart” electrical grids. Additionally, there has been a rapid increase in economic activity and urbanization, which is expected to fuel the expansion of the infrastructure and construction sector [16].

## 2. Electrical Cables

### 2.1. Structure of Electric Cables

**Figure 4:**
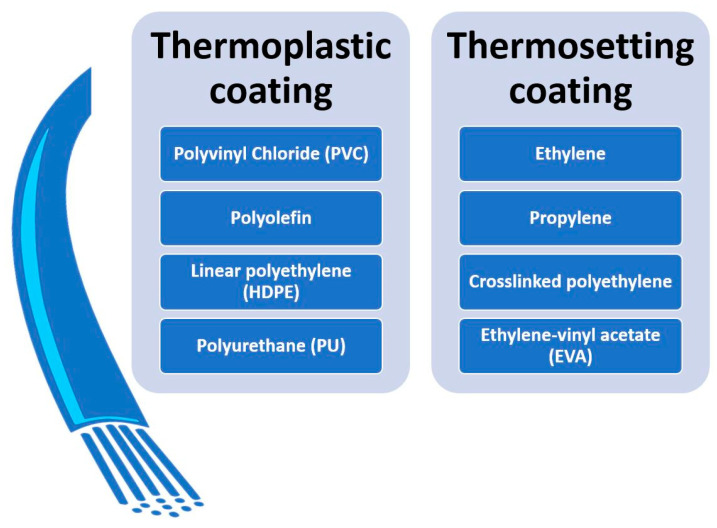
Division of the insulation layer based on Material Type. Source: own elaboration.

All products consisting of at least two layers, such as a conductor (e.g., copper or aluminum) and an insulating and/or shielding layer, are commonly regarded as cables. The construction of cables, depending on their intended use, does not differ significantly in their structure. For instance, fire-resistant cables have metal cores with an elevated melting point and a fire-resistant shielding layer, usually made of mica material. On the other hand, some cables incorporate metal shields in their structure, making them suitable for direct burial in the ground and protecting the cable core from physical damage. Auxiliary components may also be present in the conductor to secure the cable and ensure its longevity. Insulation layers, typically made of plastic material, are divided into two groups: thermoplastic and thermosetting (Figure 4).

**Figure 5:**
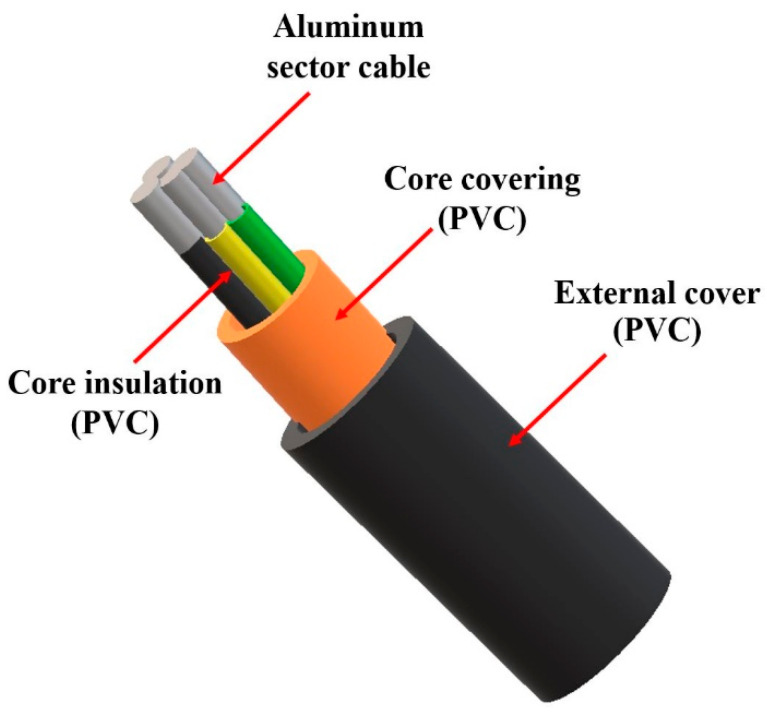
The structure of a typical low-voltage cable used in household settings. Source: own elaboration based on [17].

The structure of a low-voltage cable is presented in Figure 5.

As previously mentioned, the construction of a cable depends primarily on its purpose and usage. Additionally, the ambient temperature, air humidity, altitude above sea level, and the presence of water, corrosive substances, or mold are important factors [18]. Based on literature data, it is estimated that the conductor in cables constitutes between 52% and 70% of the total cable weight, insulation ranges from 10% to 21%, and the jacket ranges from 19% to 34% [19,20]. Table 1 illustrates several types of cables along with the estimated weight per 1 km of length.

### 2.2. Characteristics of Waste from Electrical Cables

**Figure 6:**
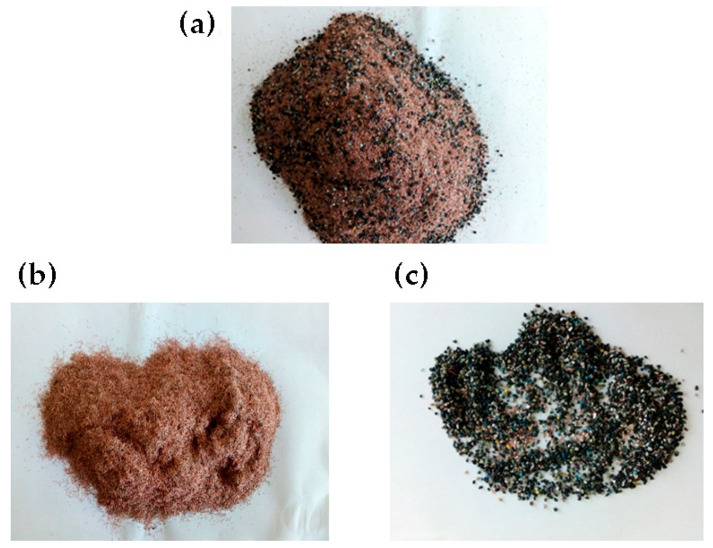
The recycled electrical cable waste: (**a**) plastic material mixed with copper, (**b**) electrical cable waste released from the cable sheath, (**c**) plastic material with small copper inclusions. Source: own resources.

Based on the gathered literature data [28,29,30,31,32,33,34,35], electrical cable waste primarily consists of copper (58.3%), polyvinyl chloride (PVC) (19.9%), and polyethylene (PE) (16%). In smaller quantities, there are polycarbonate (2.9%), silicone rubber (1.6%), steel (1.4%), and other materials (0.4%), such as cotton cord, for example. Figure 6 depicts the recycled electrical cable waste, which constitutes approximately 70% copper along with 25% PVC and polyethylene content.

Fourier-transform infrared spectroscopy (FTIR) studies [36] indicate that the cable is primarily composed of thermoplastics, such as PVC, PE, polycarbonate (PC), and silicone rubber. PVC and PE are widely used in data cables for insulating the metal parts, while PC is used in the connector segment to maintain the metal connection. The silicone rubber seal serves as a support and vibration dampener between the connector and the cable due to its low modulus. Only 29% of plastic waste from data cables constitutes the resin identification code (RIC), which is associated with the type of polymer used, while in all other samples, it is either not properly specified or gets removed during the period of use.

The average size of mixed particles ranges from 0.34 mm to 0.59 mm. After separating the plastic material, the particles can vary in size from 0.01 mm to 0.13 mm (in the case of copper) and from 0.25 mm to 0.33 mm for the plastic material. Insulation colors in plastics are assigned for identification purposes. Brown or black signifies the phase conductor, blue represents the neutral conductor, yellow-green is the protective PE conductor, and red indicates the direct current conductor. Additionally, black or blue may also indicate the negative direct current conductor [37].

## 3. Recycling of Electric Cables

### 3.1. Dismantling

**Figure 7:**
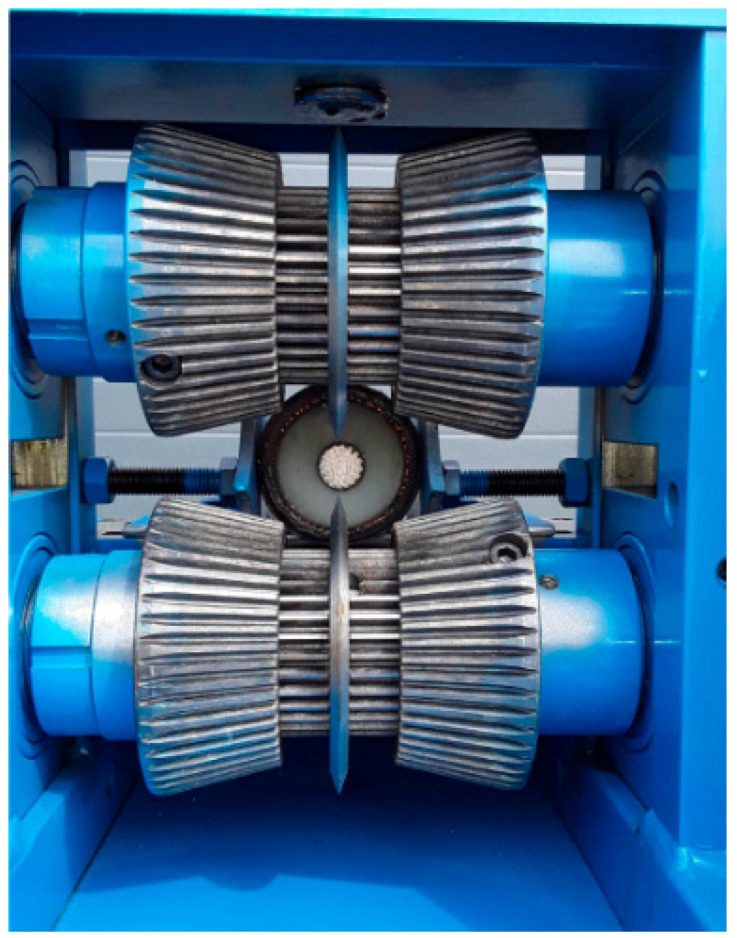
A wire stripper for separating the cable sheath from the core [38].

The dismantling of cables involves removing the insulation using a so-called wire stripper. The principle of operation of the insulation removal machine is relatively simple but can be very time-consuming depending on the type of equipment used and the amount of scrap. The dismantling process involves sorting the wires by their diameters, and then they are fed into a machine with an appropriate diameter adapted to the thickness of the wire. Subsequently, the machine, using a blade or knife, cuts the insulation while the operator feeds the next cables into the power port, and an electric motor pulls the wire. This type of machine is well-automated but has limited applicability. It can only be used to remove insulation from cables of a certain thickness with a predetermined diameter. Thicker cables cannot undergo such treatment. Therefore, recycling companies specializing in cables have significantly improved traditional insulation removal tools, making the insulation removal technology more efficient and practical. Figure 7 illustrates a wire stripper in action.

No matter how effective the insulation material separation system is, sorting is the most crucial step in the process of recovering metal from electrical cables. The properties of plastics passing through the wire stripper may deteriorate due to stress concentration during the sheath cutting.

### 3.2. The Release of Metallic and Non-Metallic Fractions (Shredding/Crushing)

**Figure 8:**
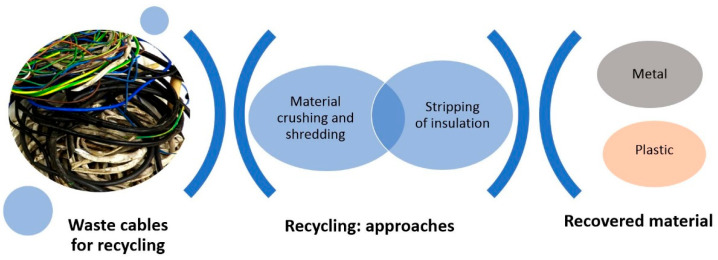
The schematic of the mechanical process of separating the cable sheath from the plastic material. Based on [39].

The process of releasing metallic fractions for large-diameter cables typically occurs in a cutting mill with grinding blades positioned around the shaft’s circumference. During grinding, most contaminants are removed from the insulation due to shear stresses generated in the mill. Contaminated dust is filtered in a three-stage process to prevent it from being released into the atmosphere. The state of dust filters is continuously monitored for the accumulation of solid particles, and they are cleaned or replaced as needed. Dust materials primarily originate from internal fillers and fibers of multicore cables. Sometimes, grinding is aided by cryogenic methods, which ensure good separation of plastic particles from metal. Figure 8 depicts the technological scheme of the mechanical processing of cables (insulation removal and crushing process).

The efficiency of wire strippers can vary depending on the following [40]:

(a) The type and thickness of insulation—thicker insulation may require more power and time to remove, affecting efficiency;
(b) The type of wires and cables—different types may require different settings and stripper speeds, impacting the work pace;
(c) Automation—a higher degree of automation, such as adjustable cutting depths, conveyor systems, or computer control, can increase the efficiency of the wire stripper;
(d) Operator experience—the skills and experience of the operator can affect work efficiency;
(e) Length of wires/cables—processing longer segments of material may require more time;
(f) Breakdowns and downtime—the frequency of breakdowns, maintenance, or technical interruptions can affect the overall machine productivity;
(g) Number and type of operators—teamwork or employing a greater number of operators can increase efficiency.

It is evident from the literature [40,41] that the efficiency of wire strippers and crushing machines often falls within the following ranges:

For wire strippers: 100–300 kg/day or 200–1000 kg/day.

For crushers: 100–1000 kg/day.

In cryogenic crushing technology (also known as the freezing process), wires are treated with an appropriate coolant (e.g., liquid nitrogen—an inert substance) to make the plastic material brittle, facilitating its crushing, while the copper core retains its strength. This process is mainly suitable for cable waste that is difficult to crush at room temperature (e.g., cables containing polypropylene (PP), low-density polyethylene (LDPE), high-density polyethylene (HDPE), or ultra-high-molecular-weight polyethylene (UHMWPE)). The efficiency of this technology is comparable to traditional methods.

### 3.3. Screening and Classification of Waste

**Figure 9:**
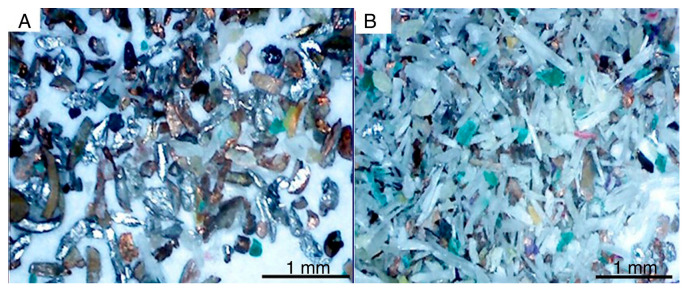
Microphotography of particle size of recycled cables: (**A**) after flotation process (**B**) before flotation process. Source: own resources.

Screening is used for classifying particles of different sizes. The mesh size of the screen can be adjusted according to the desired particle size to be separated. Screening is necessary due to the size and shape differences between metal particles and plastics. Variations in the physical characteristics of heterogeneous materials are significant. For instance, the shape of particles with a heterogeneous structure affects material properties, such as the quality of aggregates in cements and concretes [42,43,44], the catalytic properties of pharmaceuticals [45], pharmaceutical production [46], the mechanical properties in powder metallurgy [47], and the properties of industrial mineral fillers for pigments, paints, plastics, rubber, paper, etc. The significance of particle shape in many materials, including the recycling of electrical cables, is often overlooked, as particle shape influences various properties of industrial materials. Manufacturers should analyze and alter particle morphology to achieve the highest degree of metal recovery efficiency. While basic facts about particle shape can be classified as qualitative (elongation or sphericity) or quantitative (shape regularity), the latter is more important in engineering due to repeatability [48,49]. Additionally, the shape of particle material is often described using shape descriptors, which are traditional combinations of particle dimensions, such as length and width. An example of cable particle size after the flotation process is shown in Figure 9.

While the influence of particle shape on behavior is widely discussed in the literature, there is no universal approach to characterizing shape. Various analytical techniques, with different functionalities, capabilities, advantages, and limitations, from simple to advanced, are used to determine particle shape properties. However, the studied sample and the required information dictate which technique is best. Some types of cable waste are challenging to recycle using the aforementioned techniques, so chemical technology is also mentioned in the literature. Traditional chemical processing technology involves immersing solid materials in a series of extraction solutions to obtain metals, and the target metal products are then obtained through crystallization, extraction, or electrolysis of the extraction solution [50].

### 3.4. Chemical Composition Determination

When determining copper or aluminum, the well-known method utilized is ICP-OES, which stands for inductively coupled plasma optical emission spectroscopy. This method is extensively described in the literature [51,52,53,54]. However, for plastic sheaths to determine their chemical composition, various identification techniques should be employed. A single method, such as FTIR, does not provide certainty about whether the sample is pure polyethylene (PE) or polypropylene (PP) because the spectra of the wavelengths corresponding to specific functional groups can overlap. In the first stage, the plastic material is examined using organoleptic methods. This involves observing the effects accompanying the combustion of the plastic sample in a flame. The combustion method serves for the general sorting of polymer types: thermoplastics, which soften, deform, and melt under the influence of temperature, and thermosetting plastics, which do not exhibit such characteristics. During combustion tests, various characteristics are determined, including the following:

(a) Flammability and flame color;
(b) Odor and smoke development;
(c) Residue formation (e.g., ash or non-ash);
(d) Behavior of the material when exposed to fire, such as dripping or self-extinguishing properties.

These observations provide initial information about the type of plastic material used in the cable sheath. However, this method of material identification is not perfect. Human error is one of the factors contributing to its limitations. Incorrect interpretation of results and observations, such as odors (“irritating odor” or “sweet odor”), can be ambiguous and misleading [55,56,57].

The next step in material identification is the previously mentioned FTIR analysis. This analysis aims to identify the spectra of functional groups corresponding to the type of polymer. In determining compound structures, the most widely used range of the infrared spectrum is from 4000 cm^−1^ to 400 cm^−1^, which is the fundamental infrared region. In the next step of the analysis, this region is divided into four parts: (a) the range from 4000 to 2500 cm^−1^ corresponds to absorption resulting from the presence of N–H, C–H, and O-H groups in the molecule; (b) the range from 2500 to 2000 cm^−1^ corresponds to absorption resulting from the presence of groups containing triple bonds, such as alkynes and nitriles; (c) the range from 2000 to 1500 cm^−1^ primarily consists of double bond vibrations; and (d) the range below 1500 cm^−1^, known as the fingerprint region, corresponds to single bonds. Pure components such as PVC, LDPE, HDPE, and PP exhibit characteristic peaks at the following values: (a) 2972 cm^−1^ and 2910 cm^−1^, (b) 2847 cm^−1^ and 2925 cm^−1^, (c) 1465 cm^−1^, and (d) 2950 cm^−1^, respectively. The precise interpretation of IR spectra is challenging because there are many vibrational modes, both stretching and bending, within the molecule. Additionally, the same functional groups (e.g., C–O, N–H, OH) in different compounds fall within comparable wavenumber ranges.

Another step in material identification is thermogravimetric analysis. The thermal degradation of cable waste (LDPE, HDPE, PP, PE) yields three different products: condensable gases (liquid products), non-condensable gases (volatile substances), and solid residues (carbon). Gases produced during the pyrolysis of plastics consist mainly of methane (CH_4_), ethane (C_2_H_4_), and butadiene (C_4_H_6_), along with trace amounts of propane (CH_3_CH_2_CH_3_), propene (CH_3_CH=CH_2_), and n-butane (CH_3_(CH_2_)_2_CH_3_). The oil obtained after thermal degradation exhibits a range of colors, from pale yellow in LDPE to dark brown in polystyrene products. The oil obtained from LDPE and HDPE is typically a waxy product. Table 2 presents sample pyrolysis values for various plastics.

### 3.5. Gravity Separation

Gravity separation is an industrial method of separating two components, both in suspension and dry granular mixtures. The gravity separation method is one of the widely used techniques for separating plastic waste and has been extensively studied in a long-term process of practical application [59,60,61,62,63]. Gravity separation, sometimes referred to as the method of floating segregation, involves separating different types of plastic waste based on their differences in density. This method of separation is one of the oldest techniques used for plastic separation and has been significantly researched in prolonged practical application. In the case of gravity separation, the separation media needs to have varying densities that are appropriately matched to different types of plastic waste. Density differences among the waste materials themselves are utilized to identify those that float or sink in the separation media, thus achieving the goal of segregation. Traditional strategies for gravity separation of plastic waste include air separation, water separation, and centrifugal force separation [64,65,66,67,68]. PVC and PP can be separated using air separation [69,70], but it is difficult or even impossible to separate plastics (e.g., PVC and PET) with too small a density difference. In industrial production, commonly used gravity separation media include substances such as distilled water, a 55% ethanol solution, calcium chloride solution, saturated saltwater, and clean tap water. The main challenge in plastic flotation is finding effective methods for selectively wetting plastics, which can be achieved by reducing the interfacial tension between the liquid-vapor phases (referred to as the flotation gamma), chemical conditioning, and surface treatment [71,72,73]. Many reagents for the flotation separation of plastics have been studied. Table 3 presents reagents examined for plastic flotation in terms of their flotation behavior with polymeric materials. Depending on the flotation behavior of plastics in the presence of a flotation medium, appropriate reagents can be selected to separate mixtures of plastics. Additionally, these results can provide some insight into the mechanisms of action of these preparations.

### 3.6. Magnetic Separation

Magnetic separation has undergone remarkable technological advances over the past decade. As a result, new applications and design concepts for magnetic separation have emerged. This has led to a variety of highly efficient and effective magnetic separator designs. The most commonly used separators in the recycling industry are drum magnetic separators, which generate low magnetic field strengths. These separators work well for collecting ferrous materials but are ineffective for fine paramagnetic particles. High-intensity magnetic separators, which were effective at collecting fine paramagnetic particles, used electromagnetic circuits with excessive energy consumption. These separators are large, heavy machines with low capacity, typically consuming excessive energy and requiring frequent maintenance [79]. Advances in magnet materials have revolutionized the field of magnetic separation. The advent of rare-earth permanent magnets in the 1980s provided magnets with magnetic energy significantly greater than conventional ferrite magnets. Magnetic circuits made from rare-earth metals typically exhibit magnetic attraction forces 20 to 30 times greater than conventional ferrite magnets. This development has enabled the design of high-intensity magnetic circuits that operate without energy and surpass electromagnets in terms of strength and effectiveness.

**Figure 10:**
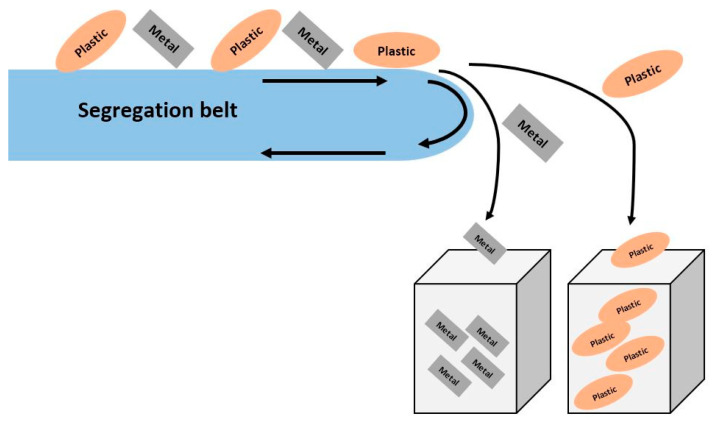
General diagram of the principle of operation of the ECS. Based on [85].

The separation of plastics from metals in cables is most commonly achieved using a device such as an ECS (eddy current separator) [80,81,82,83,84]. An ECS consists of a conveyor belt system and a magnetic drum, as shown in Figure 10. This drum rotates at high speed beneath the conveyor belt independently of the belt drive. Non-ferrous metals move towards the drum above the belt and fall into a separate container where the products are separated into metal and plastics.

Kang and Schoenung [86] conducted research that shows that the main criteria for ECS are material density, electrical conductivity, and the ratio of its density to electrical conductivity. Materials with a higher conductivity in relation to density can be separated more easily. In addition, they noticed that the separation factor is much higher for small particles than for larger ones.

### 3.7. Electrostatic Separation

Electrostatic separation involves the separation of charged particles, either metals or non-metals, under the influence of an electric field. The electric field exerts different forces on metal and non-metal particles, allowing for the separation goal of separating the material from the metal to be achieved. Conducting particles (metals) quickly discharge over the grounded surface of the rotating drum and are ejected outside the device into a metal collecting chamber. On the other hand, charged non-conductive particles are attracted by the electric force to the grounded electrode’s surface and move along with it. The detailed principle of operation of electrostatic separators can be found in the works of Veit et al., Dascalescu et al. and Xu et al. [87,88,89]. The operating parameters of an electrostatic separator in cable recycling are as follows [90]:

- Ionizing electrode: rotor distance = 25 cm, electrode inclination angle: 80°;
- Static electrode: rotor distance = 25 cm, inclination angle: 52.5°;
- Rotor revolutions per minute: 85;
- Voltage: 45–46 kV.

Based on the article [91], it was determined that the best copper recovery rate occurs at maximum input parameter values, namely high voltage and low rotor rotation speeds. Setting a low electrode voltage and a higher rotor rotation speed reduces the effect of attaching plastic particles. As a result, heavier plastic particles end up in the conductive fraction, reducing their recovery rate.

### 3.8. Feedstock Recycling

Another way of processing waste from cables is called feedstock recycling. By definition, feedstock recycling involves recovering the materials used to produce a particular product [92]. When it comes to cable production, one of the main materials is hydrochloric acid. These materials can be reused to create fully functional elements, and the waste generated from this method (light and heavy petrochemical fractions) can be used as additives for fuels and lubricants. This process consists of two stages: removing chlorine molecules from PVC and utilizing the remaining hydrocarbon portion. For example, the dechlorination process and chlorine recovery can be carried out using ethylene glycol and NaOH [93,94]. The resulting NaCl salt and glycol are separated using electrodialysis and reused in various processes. The obtained hydrocarbon fraction can be used in thermal processing or in fuel production processes. Currently, research on the thermal recycling of PVC focuses on obtaining chlorine, hydrogen chloride, and salts. These products are not treated as waste but as a valuable source of raw materials for further processes [95]. For economic and environmental reasons, this type of recycling should be applied to waste that cannot undergo mechanical recycling. Unfortunately, the use of complex installations, high temperatures, pressures, catalysts, and strict control of parameters limits the widespread adoption of these recycling methods. Improper thermal disposal of chlorine-containing waste, including PVC, can cause significant damage to the installation due to the corrosive properties of the resulting gas products. The formation of dioxins at inappropriate temperatures is also dangerous, which is why process control is essential. In addition, materials contained in the waste are irretrievably excluded from a closed-loop economy [96].

## 4. New Methods of Recycling Electrical Cables and Their Challenges

Modern electrical cable recycling focuses on several key aspects aimed at increasing efficiency, reducing environmental impact, and utilizing advanced technologies. The main areas of focus in modern cable recycling are the following:

(a) Separation and sorting: This area is centered on the precise separation of different cable components using electrodynamic and eddy current separators, allowing for automatic recognition and sorting of materials.
(b) Pollution management: This area places a strong emphasis on safe and environmentally friendly management of pollutants and chemicals that may be present in cable insulations. Cleaning and monitoring systems are employed to minimize the impact on the environment and the health of workers.

In accordance with applicable standards, plastic recycling processes are divided into four basic types: primary, secondary, tertiary, and quaternary. Each process is distinguished by a different mechanism, which can be categorized into mechanical, thermal, chemical, or biological recycling. All types of waste containing plastics are first subjected to sorting in recycling. Sorting is most commonly performed using automated facilities that utilize technologies based on electrostatics, infrared, fluorescence, or spectroscopy. The accompanying degradation of plastics during recycling is a physical phenomenon and can occur in the form of shredding or grinding, which can be classified as mechanical recycling [97,98].

### 4.1. Chemical Recycling

The waste consists of various types of polymers. In such cases, chemical recycling becomes more complex, mainly due to the variable properties of these polymers, including their different melting points. Electrical cable waste typically contains several types of polymers. Therefore, using a single fixed temperature is not suitable for the recycling process. Chemical recycling offers undeniable advantages, such as significant energy recovery and support for sustainable development. Because waste can comprise a mixture of polymers with different physicochemical properties, chemical recycling can be categorized into various methods: solvent-based purification, chemical depolymerization, and thermal depolymerization. Solvent-based purification involves breaking down waste materials, while chemical depolymerization is a process where chemical reactions yield monomers. Thermal depolymerization, also known as pyrolysis, breaks down polymers into monomers and reactivates them into hydrocarbons [99]. Recycling plastic waste using chemical technology allows for the production of monomers from polymers, which can then be reused and processed into valuable chemical substances. Chemical technologies are considered essential for developing a closed-loop system for plastics. Currently, there are increasing investments in this technology, which is expected to have a positive impact on waste reduction in the future. Depending on the method, chemical technologies can be categorized into catalytic and enzymatic depolymerization as well as solvolysis. Depolymerization, involving the breakdown of polymer chains, is often performed in the presence of strong acids, bases, enzymes, microorganisms, or metallic catalysts. On the other hand, alcohols or water are used in solvolysis processes, which rely on interactions that cleave hydrolyzing polymer bonds. Solvolysis methods can be further divided into hydrolysis, methanolysis, glycolysis, and aminolysis [100].

### 4.2. Technologies Utilizing Artificial Intelligence and Automation

The recycling process, despite ongoing development, often encounters issues related to the workforce. Therefore, new technologies are being sought to streamline recycling. The use of automated equipment and artificial intelligence can effectively support tasks such as sorting. Additionally, the introduction of robotics reduces labor time, increases efficiency, and minimizes hazards for workers. However, it should be noted that the implementation of artificial intelligence in recycling is relatively new and not as advanced as robotics in other industries [101]. Aschenbrenner et al. [101] point to the Finnish company ZenRobotics in their work, which can boast the first AI-based automated sorter.

The introduction of artificial intelligence-based technologies, automation, and robotics can contribute to improving recycling processes, increasing profitability, and optimizing recovery and material reprocessing operations. Automation in the form of robotics can successfully enhance recycling efficiency. This can be achieved through human–robot collaboration, where the machine operator performs only those tasks that require human skills not yet automated, while robots handle all tasks that can be automated. Such a division appears to be optimal and could be key to improving the efficiency of polymer material recovery [102].

One of the crucial stages in waste recycling is sorting. As indicated by Friedrich et al. [103], it is necessary for the sustainable development of a closed-loop economy to be supported by sorting technologies. These technologies often rely on sensors that allow for the automatic grouping of materials. Depending on their mode of operation, sensors can be categorized as surface-working or capable of penetrating a waste item and identifying its interior [103].

Kshirsagar et al. [104], in their work, describe how artificial intelligence supports robotic techniques in waste recycling. The authors provide detailed insights into industrial robot arms, which can be efficiently used in various recycling processes. Performing tasks such as grasping, moving, and sorting waste is fully automated and integrated into the system. Properly designed and adapted technology also allows for grouping waste into categories such as plastics or cardboard, grasping from multiple angles, and efficient arm movement within the robot’s workspace [104]. The collection and segregation of electronic waste can be supported by a mobile robot. Madhav et al. [105] describe such a robot capable of identifying electronic waste using transfer learning. The mobile robot is part of waste collection vehicles and operates by identifying and then segregating waste materials into different categories. This is performed in collaboration with a lifting and storage mechanism on the robot’s arm. The neural network utilized in the mobile robot enables precise material grouping (around 96%). The authors suggest that such a solution, implemented at the collection, segregation, and recycling levels, relieves human workers and can significantly reduce waste management costs [105]. Similar solutions were proposed by Mohammed et al. [106]. The authors developed an automated waste sorting and classification system based on artificial neural networks supported by nuclear fusion techniques. The project, which utilizes various digital models and machine learning, is effective and achieves high accuracy (approximately 91%) [106]. The last example of intelligent algorithm utilization is one developed and described by Ziouzios et al. [107]. Scientists devised an innovative method for material grouping on conveyor belts in waste collection facilities, which they claim is effective in over 92% of cases. The proposed system, based on convolutional neural networks and aided by a non-standard set of real-time graphical data collected during conveyor belt operations, detects and categorizes waste materials [108].

The use of artificial intelligence, automation, and robotics undoubtedly contributes to sustainable development and the efficient operation of waste management. It is likely that these new technologies will become global solutions that improve people’s quality of life, reduce costs associated with waste management, support human work, and, above all, increase and/or enhance the efficiency of waste recycling processes [108].

### 4.3. Microorganism-Utilizing Technologies

The degradation of plastics can also occur through the use of microorganisms. Some microorganisms found in landfills “feed” on plastic waste, obtaining a source of energy and carbon in the process. The result of microorganism activity on plastics is mineralization. Recently, there has been a significant involvement of microorganisms in plastic removal processes. However, this is still not entirely effective. Technologies utilizing microorganisms are considered a good alternative to traditional waste management methods, with a positive impact on the environment. Additionally, as indicated by Basak and Meena [109], surprisingly good results can be achieved by combining microorganism-based approaches with physicochemical methods.

One advantage and superiority of microorganisms compared to other methods used to deal with plastic waste is their easy adaptation to the environment. This is mainly possible due to the existence of various strains with varying properties and viability. Among the most popular strains of microorganisms involved in plastic degradation are *Staphylococcus*, *Bacillus*, *Pseudomonas*, and *Ideonella* [110,111]. Interesting research regarding the participation of *Pseudomonas* bacterial strains in the biodegradation of low-density polyethylene (LDPE) plastic was presented by Kyaw et al. [112]. The study observed that different strains of *Pseudomonas* successfully degrade LDPE films, as confirmed by analyses of sample mass changes, mechanical and surface changes, and spectroscopic analyses [112]. The biodegradation of microplastics was also tested against strains of *Achromobacter denitrificans* bacteria, as described by Rad et al. [113]. Degradation studies were conducted for pre-treated plastics: polyvinyl chloride (PVC) and low-density polyethylene (LDPE). Based on the results, the authors concluded that the isolated *Achromobacter denitrificans* bacterial strains successfully enhance the biodegradation of PVC and LDPE, as confirmed by structural, weight, and surface analyses [113]. Khan et al. [114] analyzed the biodegradation of LDPE waste in Indian landfills using the fungus *Penicillium citrinum*. The observation and identification of degradation processes were performed through the study of sample mass loss and spectroscopic, microscopic, and thermogravimetric analyses. The analyses revealed changes in the mass and pH of the material, structural changes in functional groups, and the occurrence of LDPE depolymerization. The authors reported that these studies are innovative due to the high efficiency of the used fungi in LDPE degradation, which had not been described before [114]. A strain of *Bacillus cereus* bacteria isolated from cow dung was used for the degradation of high-density polyethylene (HDPE) [115]. The research proved to be successful, demonstrating the breakdown of HDPE. Given the availability of the material (animal dung) from which these bacterial strains can be isolated, it can be considered an easy and environmentally friendly process [115].

The use of microorganisms for recycling plastics is a promising method that supports sustainable development and electronic waste recycling technologies. However, it is important to note that difficulties also arise in these processes. One of the challenges is that the effectiveness and efficiency of microorganisms depend on the type of plastic and its properties. Additionally, it is important to ensure that the degradation does not lead to the formation of toxic products harmful to humans and the environment [116].

## 5. Conclusions

This article discusses known methods of recycling cables and contemporary development trends. Electric cable recycling is subject to continuous improvement. The goal of these improvements is to enhance the quality of products obtained from waste materials. The more complex the process, the higher the required price of the recovered product. In recent years, mechanical recycling has been the dominant method. This technology is simple, cost-effective, has lower material losses, and offers significant adaptability. Advancements in artificial intelligence have also benefited this technology, particularly in improving recycling efficiency, such as in granule sorting. However, mechanical recycling also has its limitations, including high energy consumption and significant dust and noise pollution.

Chemical recycling still faces challenges, primarily related to the removal of heteroatoms such as halogens, sulfur, and nitrogen, which make the production of oil from these sources costly [117]. Chemical technology requires the use of a large quantity of solvents/leaching solutions, potentially leading to the generation of waste that needs disposal. In the case of technologies utilizing living organisms, isolated bacterial strains are the most promising. However, their main challenge lies in achieving industrial-scale processing. Additionally, the world is moving towards a closed-loop economy, and the profitability of such recycling is very low, although not impossible. Investments in this field may only be justified for environmental reasons.

## Figures and Tables

**Table 1:** Selected cables types, their construction and application [21,22,23,24,25,26,27].

Cable	Type	Construction	Application	Ref.
Phelps Dodge Cable	60227 IEC 01	Insulation: PVC. Core: annealed copper (stranded or solid).	Building wiring.	[21,22,23,24,25,26,27]
	60227 IEC 10	Insulation: PVC. Core: annealed copper (stranded or solid). Depending on requirements can be concentric or compact.	Surface mounting exposed. Permissible wet and dry conditions.	
	Medium Voltage Crosslinked Polyethylene	Insulation: crosslinked polyethylene. Core: copper-stranded wire, metallic shield.	Cabling of urban networks, large agglomerations. Possible use in underground conditions.	
	0.6/1 (1.2) kV Fire Resistant Low and Halogen Free	Insulation: crosslinked polyethylene. Core: two variants—copper wire round concentric or round compact.	Provision of energy for air canal, cable resources, underground.	
	CV-AWA	Insulation: crosslinked polyethylene. Core: two variants—concentric stranded annealed copper or compact round stranded annealed copper.	Providing energy in dry and wet conditions. For air canals and underground excavations.	
	Medium Voltage Crosslinked Polyethylene Cable	Insulation: crosslinked polyethylene. Core: round and compact copper wire.	Urban networks, ducts, underground. Aerial installations.	
	AL-PE Sheathed Cable	Insulation: PP or PE. Core: solid annealed copper.	Distribution and telephone lines.	

**Table 2:** Pyrolysis of different types of plastic [58].

Type of Material	Temp. [°C]	Liquid [%]	Char [%]	Gases [%]	Density at 20 °C, [g/cm^3^]
LDPE	280–420	92,16	2.1%	35	0.7821
HDPE	310–450	90,06	1.6	48	0.7962
PP	260–430	89,98	3.65	45	0.7816
PS	260–330	82,04	16.07	64	0.9127

**Table 3:** Flotation reagents used in mixture of cable plastic (PVC, PE, PC). Source: own elaboration based on [74,75,76,77,78].

Reagent	Medium Content (Frother)	Configuration of Use Mixture Plastic	Reagent Concentration [% vol]	Flotation Recovery [%]
Methanol	Methyl Isobutyl Carbinol (190 mg/L)	PET and PVC	5	95 PET
Alkyl	Methyl Isobutyl Carbinol with NaOH, HCl pH regulator, pH = 10	PVC, PC	n/a	97 PC
Calcium lignosulfonate	Pine oil (three to five drops), pH = 12	PVC and PET	0.03	95 PVC
Epoxidized linseed oil (ELO)	Methyl Isobutyl Carbinol, pH = 12.4	PET, PVC	0.03	93 PET

## Data Availability

Not applicable.
